# The Double-Row Suture Technique: A Better Option for the Treatment of Haglund Syndrome

**DOI:** 10.1155/2016/1895948

**Published:** 2016-12-18

**Authors:** Yiqiu Jiang, Yang Li, Tianqi Tao, Wang Li, Kaibin Zhang, Jianchao Gui, Yong Ma

**Affiliations:** ^1^Nanjing University of Chinese Medicine, Nanjing, China; ^2^Department of Orthopedics, Nanjing First Hospital, Nanjing Medical University, Nanjing, China

## Abstract

*Purpose*. The purpose of this study is to investigate whether double-row suture technique is a better option for the treatment of Haglund syndrome than single-row suture technique regarding the surgical outcomes.* Methods*. Thirty-two patients with Haglund syndrome were recruited in this study. Patients were divided into Group 1 (treated with single-row suture technique) and Group 2 (treated with double-row suture technique). There were 16 patients in each group. The AOFAS-ankle-hindfoot scale, VISA-A scores, and Arner-Lindholm standard were used to assess the clinical outcomes. The pre- and postoperative X-rays were used to assess the radiological outcome.* Results*. Both AOFAS-ankle-hindfoot scale score and VISA-A score had varying degrees of improvement in both groups. In latest follow-up assessment, the Arner-Lindholm standard investigation showed there were 7 excellent, 7 good, and 2 bad outcomes in Group 1 and 12 excellent and 4 good outcomes in Group 2. In Group 2 patients, there were no more posterosuperior bony prominence of the calcaneum in post-op X-rays and there were no recurrent cases. The ankle-related scale score was statistically significantly higher in Group 2 than in Group 1 (*P* = 0.029).* Conclusion*. The double-row suture technique seems to be a better option to treat Haglund syndrome than single-row suture technique.

## 1. Introduction

Posterior heel pain is a common presentation in outpatient clinics and there are many different causes [1]. In 1928, Swedish orthopedic surgeon Haglund firstly described a posterior heel pain caused by a prominent posterosuperior corner of the calcaneus in combination with wearing a rigid low-back shoe [2]. This report made people realize such special posterior heel disease, which we called Haglund syndrome.

Actually, Haglund syndrome is an enlargement of the posterosuperior prominence of the calcaneus, which is frequently associated with insertional Achilles tendinitis, bursal projection, and Achilles bursitis [3–5]. Haglund syndrome can cause mechanical impingement to the retrocalcaneal bursa and Achilles tendon. Patients with the syndrome will present with posterior heel pain and pain on passive ankle motion. Haglund syndrome can also induce inflammation and the degeneration of the Achilles tendon [6, 7], because of the abnormal high pressure between the bursal projection of calcaneus, the Achilles tendon, and the bursal impingement of the Achilles. If there is concomitant immediate reverse tension, Haglund syndrome may even cause acute Achilles tendon rupture. Unfortunately its distinct pathogenesis is still unknown. Haglund syndrome is also called “pump bump” disease because the rigid back of pump-style shoes could create pressure that stimulate the enlarged prominence during walking [8].

Haglund syndrome can be treated conservatively or surgically. Conservative treatment included the avoidance of rigid heel counter shoes, use of heel cushions, softer uppers or pads for elevation of the heel, activity modification, or local block treatment. Medication included nonsteroidal anti-inflammatory drugs or corticosteroid injection into retrocalcaneal bursa are also recommended for acute cases. However direct intratendinous steroid injections might weaken the tendon and cause tendon rupture [9]. For many cases, the effectiveness of the conservative management is low; moreover there is higher recurrent rate in conservative treatment group [10, 11]; those patients who fail conservative treatment of more than 6 months are indicated for surgical treatment [12–14].

Before 2010, a traditional single-row suture method was used to treat Haglund syndrome, by which 50–70% of the Achilles insertion was detached without compromising the tendon. After excision the calcified and the inflamed tendon, a suture anchor was used to reattach and repair the Achilles tendon [15]. However, the results of this operation were not always satisfactory. Its recurrence rate, the Achilles tendon instability, and residual heel pain limited its use and popularity.

Recently, we treated the Haglund syndrome with the double-row suture technique and obtained good clinical results. In this study, the clinical results, the safety, and efficacy of this procedure were analyzed to evaluate whether this method could give a better long-term result than single-row suture technique.

## 2. Materials and Methods

### 2.1. Patient Population

Thirty-two patients with Haglund syndrome from February 2008 to February 2014 were retrospectively reviewed; all MRI showed the posterosuperior calcaneal prominence or Achilles tendinitis. The detailed information of all 32 patients could refer to Table 1.

Patients were selected according to the following criteria:

1. Diagnosed as Haglund syndrome.
2. All treated surgically (either the single-row or double-row suture technique).
3. Follow-up more than 24 months.
4. Having both preoperative and postoperative X-rays and preoperative MRI done.

Exclusion criteria included the following:

1. Old injuries.
2. Patients with any kinds of inflammatory arthritis (such as rheumatoid arthritis).
3. Fracture or other concomitant disorders in the foot and ankle area.
4. Patients who had other comorbidities such as diabetes, severe heart disease, morbid obesity, or peripheral vascular disease who were also excluded to avoid severe surgical complications.

### 2.2. Surgical Procedure

During the surgical procedure, patient was in prone position with a thigh tourniquet. A longitudinal skin incision lateral to Achilles tendon was made. During the surgical procedure, we found most of the patients had degenerative change and inflammation with calcification scattered in their Achilles tendons.

**Figure 1:**
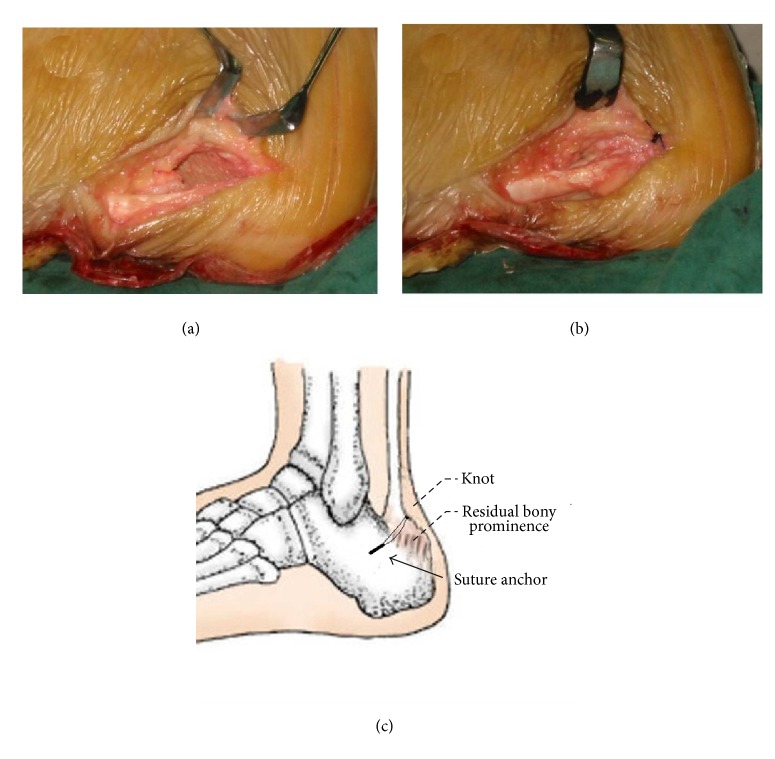
(a) 50–70% of the Achilles insertion was detached without compromising the tendon. The calcified lesions were excised. (b) A suture anchor was inserted to reattach and repair of Achilles tendon. (c) The diagram of single-row suture technique.

For Group 1, in order to ensure the continuity of Achilles tendon, only 50–70% of the Achilles insertion was detached by sharp-pointed knife. After excision of the bony prominence, the degeneration tissue, scar tissue, calcified and inflammatory tissue in the field of vision, and the detached portion of the Achilles tendon was reattached to the newly created cancellous surface of the calcaneus using one suture anchor. Two sutures connected to the anchor screw were tied with equal tension. The skin was closed with 3-0 nonabsorbable suture. In this operation, the split tendon healed in the form of point-to-point (Figure 1).

**Figure 2:**
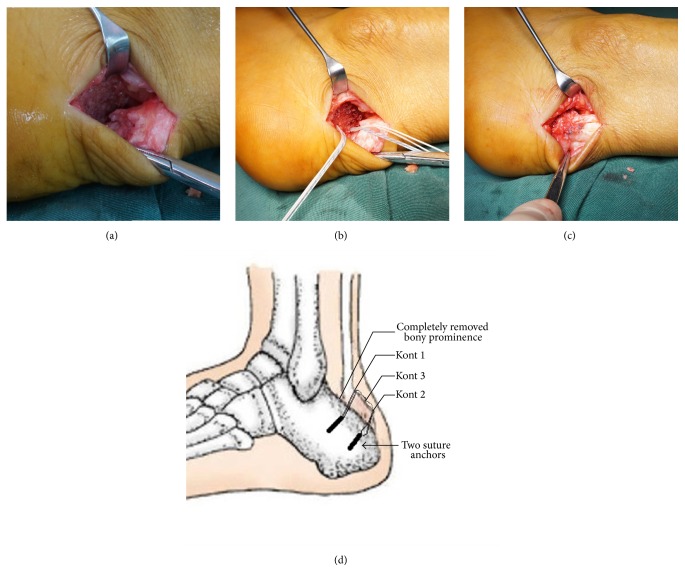
(a) Achilles tendon insertion was completely detached. The calcified tendon was completely excised. (b) Two suture anchors were inserted to reattach and repair the Achilles tendon. (c) The tendon was repaired with the double-row suture technique. (d) The diagram of double-row suture technique.

For Group 2, Achilles insertion was completely detached from the insertion site. After excising the whole bony prominence and the diseased tendon, the first suture anchor was inserted in the proximal calcaneal insertion. Krackow suture technique was used to suture the detached Achilles tendon with the 4 stitches (Knot 1). The next step was to assess the size of the posterosuperior calcaneal prominence and assess whether there was any impingement syndrome by the impaction test. Osteotome was then used to resect the posterosuperior calcaneal prominence. The second suture anchor was inserted in the distal point of calcaneum resection surface (Knot 2). The stitches passed through the terminal part of the Achilles tendon and were tied with the first 4 stitches by the double-row suture technique (Knot 3) (Figure 2). After that, the skin was closed with 3-0 nonabsorbable suture. For all Group 2 patients, no one needed flexor hallucis longus tendon transfer nor proximal V-Y advancement of the gastrocnemius fascia.

### 2.3. Postoperative Management

All patients were put on a short leg plaster cast with ankle in equinus position for 6 weeks immobilization. They were instructed on non-weight-bearing walking for 6 weeks, before full weight bearing walking was allowed. Passive dorsiflexion and active resistive plantar flexion ankle exercises were started at 6 weeks after surgery. Usually at 3 months' time, patients could participate in normal daily activities.

### 2.4. Evaluation Methods

The American Orthopaedic Foot and Ankle Society (AOFAS) ankle-hindfoot scale, the Victorian Institute of Sport Assessment-Achilles (VISA-A) scores, and Arner-Lindholm standard were used to evaluate the surgical outcomes of the patients. The preoperative and postoperative radiological features of calcaneal shapes were also assessed on the standing lateral foot X-ray.

### 2.5. Statistical Analysis

The SPSS software (version 18.0) was used for statistical analyses. The independent sample *t*-test was used for comparison of the preoperative and postoperative data. The Wilcoxon signed-rank test was used to compare the ankle-related scale score varieties between two groups. The statistical significance was set at *P* value < 0.05.

## 3. Results

All 32 patients in both groups achieved primary healing without anchor loosening, displacement, or rupture of the Achilles tendon. In Group 1, two patients had recurrent symptoms and five patients had mild residual posterior heel pain; those residual symptoms decreased patients' satisfaction. In Group 2, there were no recurrent cases. 15 patients regained normal range of motion of the ankle joint at 12 weeks and resume low impact sports at 6 mouths without posterior heel pain. One patient had delayed recovery up to one year because of the relative low threshold to pain and inadequate rehabilitation exercise. In Group 2, all patients eventually achieved satisfactory results.

The mean AOFAS ankle-hindfoot scale score, the VISA-A score, and the Arner-Lindholm standard could be refer to in Table 2. The ankle-related scale score varieties were statistically significant higher in Group 2 than in Group 1 (*P* = 0.029).

**Figure 3:**
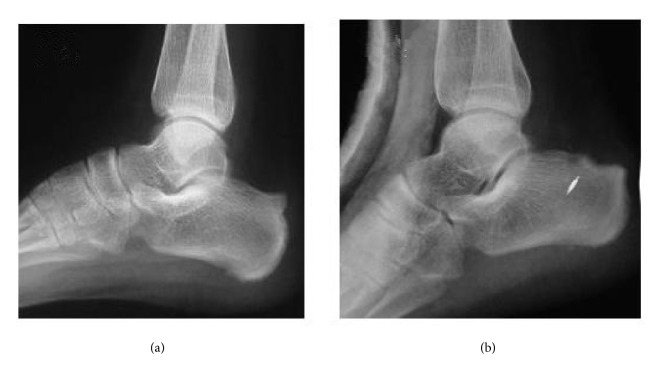
The standing lateral foot X-ray preoperatively (a) and postoperatively (b) showed the calcaneal prominence was excised.

**Figure 4:**
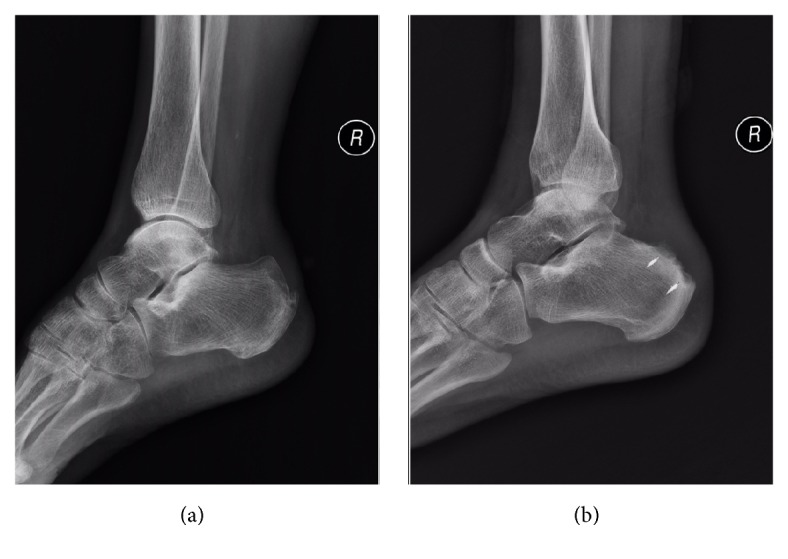
The standing lateral foot X-ray preoperatively and postoperatively showed complete excision of posterosuperior calcaneal prominence. (a) Preoperative X-ray showed obvious posterosuperior calcaneal prominence. (b) Postoperative X-ray showed the location of two suture anchors and the complete excision posterosuperior calcaneal prominence.

Radiologically, there was no posterosuperior bony prominence in the calcaneus in Group 2. And there was no impingement syndrome in all patients. The preoperative and postoperative comparison of the X-ray film could refer be to in Figures 3 and 4.

## 4. Discussion

Haglund syndrome, firstly described by Swedish orthopedic surgeon Haglund in 1928 [2], is the general description of syndrome which included posterosuperior calcaneal bony prominence, insertional Achilles tendinitis, bursal projection, and Achilles bursitis [4, 5]. From the lateral foot radiograph, the prominent calcaneal bursal projection, retrocalcaneal bursitis, and thickening of the Achilles tendon could all be seen [16]. This disease had been postulated to cause posterior heel pain resulting from mechanical impingement of the retrocalcaneal bursa [17]. Meanwhile, insertional Achilles tendinitis was regarded as an overuse phenomenon resulting in inflammation and accelerating the degeneration of the Achilles tendon at insertion site [18]. The combination of such pathologies wound seriously affected patients' walking. In worse situation the Achilles tendon might rupture with a mild trauma event.

Patients with Haglund syndrome varies in age from young to elderly, and it is more commonly seen in women [19]. The exact pathogenesis is still unknown. The possible causes include inheritance factor, injuries related to the sport, inappropriate shoe wear, and sequelae of calcaneal fractures [20]. It is usually diagnosed clinically and radiologically. The standing lateral foot radiograph is useful to assess the presence of the posterosuperior calcaneal bony prominence (Haglund deformity) [16, 21]. MRI has superior soft tissue and bone marrow signal sensitivity, which facilitate it to make diagnosis of Haglund syndrome [22], especially for those ambiguous or clinically equivocal cases.

To date, the management of Haglund syndrome included conservative and surgical treatments [23]. Conservative treatment included the avoidance of rigid heel counter shoes, use of heel cushions, softer uppers or pads for elevation of the heel, activity modification, or local block treatment. Medications including nonsteroidal anti-inflammatory drugs or corticosteroid injection into retrocalcaneal bursa are also recommended for acute cases. Although the bursitis could be controlled by these methods, the posterosuperior calcaneal bony prominence could not be removed. Such mechanical impingement causes persistent heel pain. It is controversial whether the inflammation in the bursa or tendon could be relieved by local block treatment, as the persistent inflammation in the bursa or tendon could lead to rupture of the Achilles tendon [9].

If conservative treatment failed, surgical intervention should be recommended [24–26]. It has been believed that traditional one suture anchor with single-row suture technique, by which 50–70% of the Achilles insertion was detached from the insertion, repaired, and reattached after the procedure, can improve the heel pain. However, concerning such method, inadequate bone resection could lead to recurrence of heel pain. Moreover, point-to-point tendon healing may not restore the full strength and the stability of the Achilles tendon, which may weaken it or even cause rupture of the Achilles tendon.

In view of such disease, we conclude that the treatment principles should include excision of the Haglund deformity, relieving the mechanical impingement, and restoring the continuity of Achilles tendon [13]. Comparing these principles with the rotator cuff repair technique [27], we derived a concept of double-row suture technique, which obtained good clinical outcomes and high patients' satisfaction. During the operation, the first step was to excise the degenerative and scar tissue, thorough debridement of the calcified and the inflamed tendon. Secondly, two suture anchors were inserted in the proximal and distal point of Haglund deformity bone resection surface. The detached ends of Achilles tendon were repaired by the sliding suture of the anchor. Thirdly, through the double-row suture technique the sutures on each anchor were tied over with the another. With such repair method, it provided larger contact area between tendon and bone surface and promotes the healing of the Achilles tendon.

In this study, we obtain long-term satisfactory outcomes in an average follow-up period of 3.5 years. Compared to the single-row suture group, all patients obtained primary healing. There are no significant complications, such as spur recurrence or residual heel pain. An analysis of the various ankle-related scores consists of the American Orthopedic Foot and Ankle Society (AOFAS) ankle-hindfoot scale [28], Victorian Institute of Sport Assessment-Achilles (VISA-A) scores [29], and Arner-Lindholm standard [30] which were also conducted to evaluate the clinical effect. All these scores obtained satisfactory results. The AOFAS ankle-hindfoot scale score improved from 59.2 ± 6.7 preoperatively to 91.1 ± 4.2 at the latest follow-up visit, and the VISA-A score improved from 50.6 ± 3.2 to 90.6 ± 3.4. The Arner-Lindholm standard investigation at the latest follow-up visit showed 11 excellent, 5 good, and no bad outcomes. Postoperative X-rays showed complete excision of the Haglund deformity. Whilst in the single-row suture group, the Arner-Lindholm standard investigation at the latest follow-up visit showed 7 excellent, 7 good, and 2 bad outcomes. Two of the patients had recurrence and five patients had residual posterior heel pain.

Using our technique, we can overcome the previous complications. The complete excision of posterosuperior calcaneal bony prominence (Haglund deformity) can effectively relieve the heel pain and prevent the recurrence. The larger contact surface between tendon and bone will facilitate tendon healing and stability. The shorter period of immobilization (plaster cast after surgery) allowed early functional exercise and reduced the joint stiffness. Early activity also maintains gastrocnemius muscle capacity and minimizes the plantar flexor muscle strength deficit. Therefore, the double-row suture technique can improve clinical outcome of Haglund syndrome.

## 5. Conclusion

For those patients with the Haglund syndrome, the double-row suture technique could be a better option for its satisfactory surgical outcomes than traditional single-row suture technique.

## Figures and Tables

**Table 1:** The detailed information of the 32 patients.

Group	Gender		Average age	Right or left		Mean follow-up duration
	Male	Female		Right	Left	
Group 1	06	10	50.6 ± 3 years (range, 21 to 59 years)	8 (50.0%)	8 (50.0%)	3.5 ± 0.8 years (range, 24 to 60 months)
Group 2	05	11	52.1 ± 2 years (range, 33 to 68 years)	9 (56.3%)	7 (43.7%)	3.5 ± 0.5 years (range, 24 to 60 months)

Group 1: the patients had traditional single-row suture method.

Group 2: the patients had double-row suture technique.

**Table 2:** Comparison of functional scores pre- and postoperatively in 2 groups (*N* = 16 patients in each group).

	Scale	Preoperative score	Latest follow-up score	*P*value^*∗*^
Group 1	AOFAS ankle-hindfoot scale score	56.1 ± 4.1	81.3 ± 6.5	0.0441
	VISA-A score	52.6 ± 5.2	84.1 ± 3.9	0.0408
	The Arner-Lindholm standard		7 excellent, 7 good, 2 bad	
	Recurrence rate		2 (12.5%)	
	Residual heel pain		5 (31.3%)	

Group 2	AOFAS ankle-hindfoot scale score	59.2 ± 6.7	91.1 ± 4.2	0.0228
	VISA-A score	50.6 ± 3.2	90.6 ± 3.4	0.0158
	The Arner-Lindholm standard		11 excellent, 5 good, 0 bad	
	Recurrence rate		0	
	Residual heel pain		0	

AOFAS: American Orthopaedic Foot and Ankle Society.

VISA-A: Victorian Institute of Sport Assessment-Achilles.

Data presented as mean ± standard deviation.

Group 1: the patients had traditional single-row suture technique.

Group 2: the patients had double-row suture technique.

^*∗*^Independent sample *t*-test.
